# Proteome of *Aedes aegypti* in response to infection and coinfection with microsporidian parasites

**DOI:** 10.1002/ece3.199

**Published:** 2012-04

**Authors:** Alison B Duncan, Philip Agnew, Valérie Noel, Edith Demettre, Martial Seveno, Jean-Paul Brizard, Yannis Michalakis

**Affiliations:** 1 Maladies Infectieuses et Vecteurs: Ecologie, Génétique, Evolution et Contrôle (MIVEGEC)UMR5290 CNRS-IRD-Université de Montpellier I, Université de Montpellier II, 911 avenue Agropolis, 34394 Montpellier, France; 2 Institut de Génomique FonctionnelleCNRS UMR 5203,INSERM U661, Universités Montpellier I & II, Plate-forme de Protéomique Fonctionnelle CNRSUMS BioCampus 3426, 34094 Montpellier, France; 3 UMR IRD-CNRS-UPVD 5096, Institut de Recherche pour le Développement (IRD)Montpellier, France

**Keywords:** *Aedes aegypti*, coinfection, dengue, *Edhazardia aedis*, proteome, *Vavraia culicis*

## Abstract

Hosts are frequently infected with more than one parasite or pathogen at any one time, but little is known as to how they respond to multiple immune challenges compared to those involving single infections. We investigated the proteome of *Aedes aegypti* larvae following infection with either *Edhazardia aedis* or *Vavraia culicis*, and coinfections involving both. They are both obligate intracellular parasites belonging to the phylum microsporidia and infect natural populations of *Ae. aegypti*. The results found some proteins only showing modified abundance in response to infections involving *E. aedis*, while others were only differentially abundant when infections involved *V. culicis*. Some proteins only responded with modified abundance to the coinfection condition, while others were differentially abundant in response to all three types of infection. As time since infection increased, the response to each of the single parasite infections diverged, while the response to the *E. aedis* and coinfection treatments converged. Some of the proteins differentially abundant in response to infection were identified. They included two vacuolar ATPases, proteins known to have a role in determining the infection success of intracellular parasites. This result suggests microsporidia could influence the infection success of other intracellular pathogens infecting vector species of mosquito, including viruses, *Plasmodium* and *Wolbachia*.

## Introduction

An important factor determining an organism's fitness is its ability to defend itself against attack from parasites or pathogens. Our understanding of how immune systems function has advanced with the advent of technology allowing the proximate mechanisms involved to be studied at the cellular or molecular level, for example, transcriptome and proteome techniques. Much of the progress made in this area has been based on how hosts respond to challenges from individual species of parasite or pathogen. However, hosts are frequently exposed to simultaneous immune challenges from different sources, and have to deal with infections involving more than one infectious agent (Petney and Andrews 1998). At present, little is known as to how hosts respond to the challenges posed by multiple infections, as opposed to those from single infections.

Coinfections increase the potential number of direct or indirect interactions capable of influencing the fitness of each organism involved. An applied context where such interactions are relevant is when the ability of one pathogen to infect and transmit from a vector is altered by the presence of a coinfecting pathogen. The infection and transmission success of some viruses can be enhanced when coinfecting mosquitoes with *Brugia* nematodes (Turell et al. 1984; Vaughan and Turell 1996). In contrast, the ability of mosquitoes to transmit other viral diseases (including dengue and chikungunya) or malarial parasites (*Plasmodium* spp.) can be decreased when mosquitoes are coinfected with entomopathogenic fungi, for example, *Metarhizium* (Blanford et al. 2005; Scholte et al. 2005), or *Wolbachia* bacteria, (Moreira et al. 2009; Bian et al. 2010). In some cases, the altered infection and transmission success of the human pathogen was influenced by the response of the mosquito immune system to the coinfecting pathogen (Moreira et al. 2009).

In this study, we compared the proteome of the mosquito *Aedes aegypti* in response to single infections and coinfections involving the obligate intracellular microsporidian parasites *Vavraia culicis* and *Edhazardia aedis*. The microsporidia are among the most common pathogens infecting natural populations of mosquitoes (Castillo 1980). Mosquitoes will encounter situations where they are challenged by one or more microsporidia and also coinfections involving a microsporidian parasite and other types of pathogens. Furthermore, it is already known *V. culicis* elicits immune responses from *Ae. aegypti* larvae (Biron et al. 2005), and its infections can reduce the establishment and development of *Plasmodium* infections in mosquitoes (Bano 1958; Bargielowski and Koella 2009).

The proteome of infected and coinfected mosquito larvae at five and 15 days postinfection were compared to that of uninfected larvae. In each case, there was also a corresponding treatment in which infected and uninfected larvae experienced 5 h of hypoxia prior to sampling. This treatment was performed to help distinguish between responses specifically related to infection from those induced by a stressful nonimmunological challenge. The observed patterns of protein abundance were interpreted in terms of how specific the host response was to each type of infection and to what extent it was shared among the different infection treatments. The pattern of how these responses changed between the two sampling periods was also addressed. Our results are compared with those from other studies involving mosquitoes or other Diptera subject to immune challenge or infection.

## Methods

### Mosquitoes

*Aedes aegypti* occurs throughout the tropics and subtropics where it is the principle vector of dengue and yellow fever. Its larvae are found in a variety of natural and artificial containers holding clean fresh water. Larval developmental time varies from six days to more than one month depending on environmental conditions (Southwood et al. 1972). The *Ae. aegypti* strain used in this study derives from a large number of eggs originally collected in Tingua, Brazil by Ricardo Lorenço de Oliveira of the Instituto Oswaldo Cruz (Rio de Janeiro, Brazil) and has since been maintained in standardized laboratory conditions with more than 3000 reproductive adults in each generation. The population was kept at 23°C (±3°C), 75% (±5%) humidity with a 12 h:12 h light:dark photoperiod. At the time of this experiment, the population had reproduced for 20 generations in our laboratory.

### Microsporidia

The two species of microsporidia used in this experiment, *V. culicis* and *E. aedis*, are both natural parasites of *Ae. aegypti*. *Vavraia culicis* has been reported from several genera of mosquitoes (Weiser and Coluzzi 1972), while *E. aedis* has only been reported from *Ae. aegypti* (Andreadis 1994). In both cases, horizontal transmission occurs when larvae ingest spores along with their food. Conditions in the mid-gut stimulate the germination of spores, resulting in the expulsion of a long hollow polar tube thought capable of traversing the peritrophic matrix and piercing host cells lining the gut. Contents of the spore pass down the tube directly into the host cell cytoplasm, thus initiating infection.

Once within a host cell's cytoplasm, *V. culicis* undergoes a series of developmental stages that culminate in the production of a single type of uninucleate spore. These spores are infectious to neighboring cells within the host or to other larvae once released into an aquatic environment, following the death of the host as a larva or pupa.

In the case of *E. aedis*, larvae ingest uninucleate spores along with their food and these germinate to infect cells lining the host mid-gut. An initial sequence of development leads to the production of binucleate spores. These spores are responsible for the transmission of infection to other cells within the same host (autoinfection), with oenocytes thought to be their main target (Johnson et al. 1997). Infected oenocytes circulate in the hemocoel and are often found in close physical proximity to a female mosquito's reproductive tissues. A second type of binucleate spore is produced within infected cells and is responsible for vertical (transovarial) transmission when they germinate and inject their contents into developing oocytes (Johnson et al. 1997). Further development occurs in the fat body of infected offspring and includes the production of uninucleate spores. The accumulation of these uninucleate spores is associated with the larval and pupal mortality, after which they are released into the aquatic environment. The life cycle is completed when they infect larvae ingesting them. The life cycle of *E. aedis* as described above includes a sequence of horizontal and then vertical transmission in sequential generations of its host. Repeated sequences of either horizontal or vertical transmission may also occur if horizontally infected larvae fail to emerge as adults, or if vertically infected females survive to adulthood (Becnel et al. 1989; Agnew and Koella 1999).

Although the two parasites initially exploit the same host tissues, they subsequently diverge as their life cycles progress and they exploit different host tissues for different purposes.

The *V. culicis* and *E. aedis* materials used in this experiment were originally provided by Dr. J. J. Becnel of the USDA/ARS (Gainesville, FL), with *V. culicis* being the Florida isolate *V. culicis floridensis* (Vavra and Becnel 2007).

### Experimental procedure

The experiment investigated the effects of single infections or coinfections on the proteome of host larvae. Larvae were exposed to different infection conditions and then sampled when either 5 or 15 days' old. In each treatment and time period, half of the larvae to be sampled experienced a period of hypoxia. This was done to help distinguish the effects of infection from responses to stress of a more general and nonimmunologic nature.

Larvae were hatched from eggs in a reduced atmospheric environment and groups of 60 larvae were transferred to petri dishes (55-mm diameter) containing mineral water (Eau de Source, Carrefour, France). Larvae were exposed to parasites on the same day as hatching. Each petri dish was randomly assigned to one of four infection treatments; (1) *V. culicis* only, (2) *E. aedis* only, (3) *V. culicis* and *E. aedis* together, and (4) no infection. There were four replicate Petri dishes per infection treatment. Each Petri dish assigned to infection treatments (1) and (3) received 1.2 × 10^6^ spores of *V. culicis* suspended in 1 mL of mineral water and those assigned to treatments (2) and (3) received 6 × 10^3^ spores of *E. aedis* suspended in 1 mL of mineral water. These spore densities yield >90% infection rates in similar experimental conditions (Duncan et al., unpubl. ms.). Each Petri dish assigned to treatments (1) and (2) also received 1 mL of mineral water, whereas those assigned to treatment (4) received 2 × 1 mL of mineral water to equalize the overall volumes. All Petri dishes received 3.6 mg of Tetramin fish food. After 24 h (day 1), larvae from the four Petri dishes within each treatment were rinsed and combined together.

For each infection, hypoxia, and sampling treatment, 60 *Drosophila* tubes (20 × 90 mm) containing 4 mL of mineral water were prepared and a single larva was added to each. Tubes were arranged in racks containing four rows of 10 tubes. Each row within a rack corresponded to a particular treatment. Racks were blocked to contain a single row of each infection, hypoxia, and sampling treatment, with rows within each block being assigned at random. To each tube, 0.8 mg of Tetramin fish food dissolved in 1 mL of mineral water was added. In these conditions, this amount of food is enough for most individuals to reach the fourth larval instar, but not enough to initiate pupation (Bedhomme et al. 2004), thus mimicking developmental conditions often encountered in natural conditions (Southwood et al. 1972). The position of racks within the room was rotated daily to minimize position effects. The room was maintained at 25°C (±3°C), 75% (±5%) humidity with a 12 h:12 h light:dark photoperiod.

Larvae were sampled from each treatment group on two occasions, day 5 and day 15. These two ages were chosen to investigate the effects on the host proteome at relatively early and late stages of infection. On each sampling day, a layer of vegetable oil (2 mL) was added to the tubes assigned to the hypoxia group 5 h before sampling. This prevented larvae from having access to atmospheric oxygen, thus causing stress, without killing them. Sampled larvae were stored at –80°C.

After sampling, larvae were individually suspended in 100 µl of the extraction/precipitation buffer (10% TCA in acetone). Larvae were crushed individually in their 1.5-mL Eppendorf tubes and 25 µl was removed for DNA extraction. The remainder of the sample was stored at –20°C until required for protein labeling and sampling. The sample for DNA extraction was centrifuged for 6 min at 13,000 ×*g*. The supernatant was poured off to remove acetone and the extraction done on DNA in the pellet according to manufacturers’ instructions using DNeasy Blood and Tissue Kit (Qiagen). Primers to confirm infection by each parasite were designed using primer 3 (Rozen and Skaletsky 2000) Primers VC16SF 5′-ggcggtagtaaggagacgtg-3′ and VC16SR 5′-cttgttacgacttgtatca-3′ were used to amplify a 968 bp fragment of the 16S gene in *V. culicis*. For *E. aedis* primers FQEA187 5′-agtgcgtaccgaggctataac-3′ and RQEA310 5′-ctcaacgttcattgggtaagtttc-3′ were used to amplify a 123 bp segment of the small subunit ribosomal RNA gene.

Polymerase chain reaction (PCR) for both parasites was performed on a Mastercycler Eppendorf. The PCR mix for *V. culicis* contained 2 µl of DNA added to a reaction mix containing 5 µl of Multiplex PCR Mix (Qiagen), 1 µl of Solution Q (Qiagen), 0.2 µl of each primer at a concentration of 10 µM^–1^, and 1.6 µl of deionized water. For *E. aedis*, 6 µl of DNA was added to 2 µl of PCR reaction buffer (Qiagen), 0.4 µl of dNTP Mix (Promega), 0.5 µl of each primer at a concentration of 10 µM^–1^, 0.2 µl of Taq DNA Polymerase (Roche), and 10.4 µl of deionized water. The amplification profile for *V. culicis* comprised of an initial denaturation at 94°C for 15 min followed by 35 cycles of denaturation at 94°C for 10 sec, annealing at 57°C for 1 min and 30 sec, and extension at 72°C for 1 min, with final extension at 60°C for 10 min. The amplification profile for *E. aedis* comprised an initial denaturation at 92°C for 2 min followed by 45 cycles of denaturation at 92°C for 30 sec, annealing at 60°C for 30 sec, and extension at 72°C for 45 sec, with a final extension for 5 min at 72°C. For identification of both parasites, 10 µl of the amplified PCR product was mixed with 2 µl of Ez-vision (Amresco) dye (10× dilution). *Vavraia culicis* PCR products were electrophoresized on a 1.5% agarose gel and *E. aedis* on a 2% agarose gel and the presence of each parasite identified under ultraviolet light.

Only larvae that were confirmed to be infected with *E. aedis* from the single *E. aedis* and the coinfection treatments, from sampling days 5 and 15, were included in samples for protein sampling and labeling. *Vavraia culicis* infection could only be confirmed for mosquitoes collected on day 15 due to insufficient DNA amplification from larvae collected on day 5. Infection levels for all treatments where infection could be confirmed are reported in Appendix A1. The potential inclusion of uninfected individuals would make our estimates of the effects of *V. culicis* on day 5 conservative as differential protein abundance between treatments would become less obvious.

### Preparation of protein samples and labeling

Larvae from each treatment group, already crushed and suspended in 100 µl of the extraction/precipitation buffer (10% TCA in acetone) were combined, where possible, in groups of 10 to form three replicates per treatment group. Larvae from the coinfection treatments sampled on day 15 had to be combined in groups of seven for those exposed to hypoxia and nine for those not exposed to hypoxia due to limited numbers of infected individuals in these groups. Samples were centrifuged at 30,000 ×*g* for 15 min at 3°C, the supernatants removed, and the samples suspended in a wash buffer (10% acetone). This step was repeated three times before being suspended in 36 µl of solubilization buffer (7 M urea, 2 M thiourea, 4% CHAPS 30 mM Tris, 0.5% Triton ×100, adjusted to pH 8.5) overnight at room temperature with agitation. The samples were centrifuged again (15 min at 30,000 ×*g*) and protein content determined after a 10-fold water dilution using the Coomassie Protein Assay.

Proteins were labeled according to the Ettan DIGE minimal labeling protocol (GE Healthcare). For each sample, 26 µg of sample was labeled with 200 pmole of CyDye™. The dye swap technique was used to label samples to control for any dye-specific effects that might result from preferential labeling, or different fluorescence characteristics of the gel or glass plates. A total of 24 gels were run. Single *E. aedis* infection treatments were always run against the coinfection treatments and the single *V. culicis* infection treatments against the uninfected control group. Samples containing larvae sampled on either day 5 or 15 and that experienced the same hypoxia treatment were always run together. An internal standard comprising a 13-µg aliquot from each sample was labeled with Cy2. The internal standard controlled for variation observed between gels.

### Protein separation by 2-Dimensional Electrophoresis

For isoelectric focusing (IEF), the internal standard labeled with Cy2, one sample labeled with Cy3, and another with Cy5 were mixed in a rehydration solution (7 M urea, 2 M thiourea, 4% CHAPS, 0.5% Triton X100, 1.2% Destreak Reagent [GE Healthcare] and 0.75% immobilisation pH gradient (IPG) buffer pH 3–10). The solubilized proteins were loaded onto 24-cm IPG strips, pH 3–10, NL (GE Healthcare). Following 14 h of passive rehydration, IEF was performed using an IPGphor apparatus (GE Healthcare) as follows: 3 h at 100 V, 3 h gradient to 1000 V, 4 h gradient to 8000 V, and 8000 V constant to reach a total of 50,000 Vh. After IEF, the strips were incubated for 15 min in equilibration buffer (6 M urea, 300 mM Tris pH 8.8, 0.2% SDS, 30% glycerol, 1% DTT) followed by 15 min in equilibration buffer where DTT was replaced with 2% iodoacetamide. The equilibrated IPG strips were placed on top of an SDS-polyacrylamide gel (12.5% acrylamide) and sealed with 1% agarose. The electrophoresis was performed using an Ettan Dalt Twelve (GE Healthcare) at 17°C and 17 mA/gel.

### Gel imaging, image analysis

Two-dimensional (2D) gels were scanned with a Typhoon 9400 set according to manufacturers’ instructions (GE Healthcare). Images were analyzed using the Progenesis SameSpots software (Nonlinear Dynamics). After alignment, detection, and normalization, the raw data were exported for statistical analysis.

### Statistical analysis

Protein abundance of spots in different treatments was analyzed with fully factorial three-way analyses of variance (ANOVAs). The factors in the ANOVA model were “day of sampling,”“hypoxia treatment,” and “infection treatment,” with the response variable being the log-normalized volume of the spot determined by the SameSpots software.

We were interested in identifying proteins with abundance profiles that were significantly different in the infected larvae to that of uninfected individuals. Furthermore, we focused on proteins where this modified abundance was specifically associated with infection and not the combined effects of infection and hypoxia. To do so, we classified the ANOVA results for each protein according to the significance of different effects in the output model, and where appropriate, tested for significant differences between infected and uninfected treatments. Only spots with a significant “day by infection” interaction and/or a significant effect of “infection” were retained. Post-hoc multiple comparison Student's *t*-tests were used to identify if spot abundance in hosts from the different infection treatments was significantly different from that of uninfected (control) hosts from the same day of sampling, or across both sampling days. As a conservative measure to protect against false-positive results (type I errors), multiple comparisons between infected and uninfected treatments were only performed if the overall ANOVA model for the spot was significant (*P*≤ 0.05). The statistical software JMP v 8.0.1 (SAS Institute Inc.) was used for these analyses. We tried to identify all proteins whose abundance was significantly modified in infected larvae.

### Protein identification: Tryspin digestion and MALDI-TOF mass spectrometer (MS) analysis

Enzymatic in-gel digestion was performed automatically (Tecan freedom evo® proteomics) according to a modified protocol described by Shevchenko et al. (1996). Briefly, protein spots were digested using 150 ng of trypsin, peptide extraction was performed using 5 sonication cycles of 2 min each, and peptides were concentrated for 1 h at 50°C in the thermoblock hotel.

Peptide samples were automatically spotted (Tecan freedom evo® proteomics): 0.5 µl of sample peptide and 0.5 µl of alpha-cyano-4-hydroxy-*trans*-cinnamic acid (the saturated solution was prepared in acetonitrile/trifluoroacetic acid, 50:0.1%, vortexed, sonicated for 30 sec, microcentrifuged for 30 sec then a one of three dilution of the supernatant was used as the matrix) were deposited on a 384-well MALDI anchorchip target using the dried droplet procedure (Karas and Hillenkamp 1988) and air dried at room temperature. Peptide samples were then desalted on the target using a 10 mM phosphate buffer and dried again at room temperature.

Analyses were performed using an UltraFlex MALDI TOF-TOF MS (Bruker Daltonics, Bremen, Germany) in reflectron mode with a 26 kV accelerating voltage and a 50 ns delayed extraction. Mass spectra were acquired in the automatic mode using the AutoXecuteTM module of FlexcontrolTM 3.0 (Bruker Daltonics) (laser [N_2_ 337 nm] power ranged from 40% to 50%, 600 shots).

Spectra were analyzed using FlexAnalysisTM 3.0 software (Bruker Daltonics) and calibrated internally with the autoproteolysis peptides of trypsin (m/z 842.51; 1045.56; 2211.10). Peptides were selected in the mass range of 900–3000 Da.

Peptide mass fingerprint identification of proteins was performed by searching against the Insecta entries of either SwissProt or TrEMBL databases (http://www.expasy.ch) using the Mascot v. 2.2 algorithm (http://www.matrixscience.com) with trypsin enzyme specificity and one trypsin missed cleavage. Carbamidomethyl was set as fixed cysteine modification and oxidation was set as variable methionine modification for searches. A mass tolerance of 50 ppm was allowed for identification. Matching peptides with one missed cleavage were considered as pertinent only when there were two consecutive basic residues or when arginine and lysine residues were in an acidic context. Mascot scores higher than 68 were considered significant (*P* < 0.05) for SwissProt and TrEMBL database interrogation. The spectra were also searched against the “Fungi” subset (Mascot score 56, *P* < 0.05) and against the “All entries” taxonomy (Mascot score 69, *P* < 0.05) of Swissprot and no proteins were identified from non-*Aedes* sources.

## Results and Discussion

The Progenesis SameSpots software (Nonlinear Dynamics) used to analyze the replicate gels for each infection and hypoxia treatment identified 646 protein spots after scanning for speckling and artifacts. A fully factorial ANOVA for the treatment effects of infection, hypoxia, and day of sampling was performed for each spot. These analyses found the abundance of 323 spots varied significantly in response to one or more treatment conditions. However, we only retained 75 spots for analysis in greater detail. These were spots with abundance profiles attributable to infection treatments only, or when an interaction between sampling day and infection treatment was observed. Proteins not retained for further analyses are categorized in Appendix A2.

### Protein abundance in relation to infection

**Figure 1:**
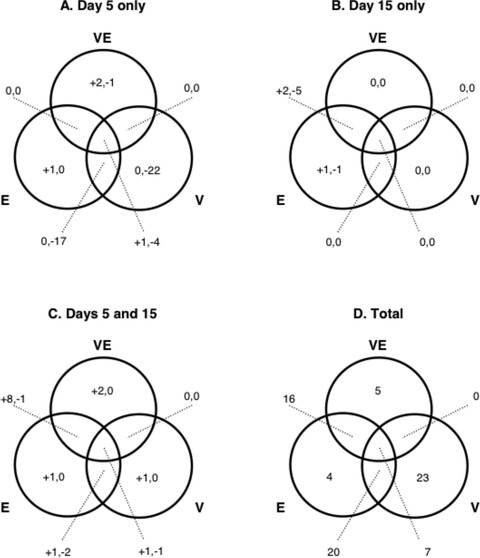
The number of proteins with modified abundance and the treatment(s) in which they were found. The numbers indicate the number of proteins from infected larvae with spot abundance significantly lower (−) or higher (+) than for uninfected larvae sampled on the same day(s). (A) Proteins only showing modified abundance in five-day-old larvae. (B) Proteins only showing modified abundance in 15-day-old larvae. (C) Proteins with modified abundance in both five- and 15-day-old larvae. (D) The absolute number of proteins with modified abundance, including both days of sampling. The “VE” label is for the coinfection treatment involving both *V. culicis* and *E. aedis*, the “E” label is for the *E. aedis* only infection treatment, and the “V” label is for the *V. culicis* only infection treatment.

The results of the analyses for the remaining 75 spots are shown in Figure 1. In total, 48 proteins showed modified abundance on day 5 only, nine had modified abundance on day 15 only, and 18 had modified abundance on both days 5 and 15. The majority of modified abundance occurred earlier in the experiment, with 64% (48/75) of proteins differentially abundant on day 5 only, compared to 12% (9/75) on day 15 only.

Spots from infected larvae tended to be smaller than those from uninfected larvae (54 smaller, 21 bigger). However, we do not focus closely on how the volume of spots changed, but rather on the fact that there was significant change in volume relative to the uninfected control. This is a conservative measure as we only have data on how much protein was present at the time of sampling and no direct evidence that an increase (or decrease) in spot volume corresponds to an increase (or decrease) in protein production. A decrease in spot volume could reveal decreased protein production or that the proteins were being metabolized at a faster rate. We first consider the general patterns of protein abundance in response to the infection treatments. We then present data on the identity of some of the proteins involved, their functional role, and how these results compare with other proteome studies in the literature.

### Patterns of protein abundance observed in the data

Here, we consider the observed patterns of protein abundance in response to the different infection treatments illustrated in Figure 1. The majority of the host response toward infection was particular to the different infection treatments. The host response common to all three infection treatments is illustrated in the zone where the three infection treatments intersect on Figure 1. Only seven of the 75 spots responded in the same way to both the single infections, and the coinfection treatment (Fig. 1D). Hence, we focus discussion on (1) how the mosquito responded to single infections of *V. culicis* (V) and *E. aedis* (E) and (2) how the coinfection treatment (VE) influenced the host proteome compared to each of the single infection treatments.

#### The specificity of the Ae. aegypti response to single infections of each parasite

The specificity of the host response toward either parasite can be assessed from the zones of Figure 1 exclusive to each parasite (i.e., that do not intersect with zones involving the other parasite). In the case of *V. culicis*, there were 23 spots fitting this description and four for *E. aedis* (Fig. 1D). Hence, part of the host response was specific to each parasite, though more so for *V. culicis* than *E. aedis*. In addition, there were 16 spots observed at the intersection of the E and VE treatments, meaning they were differentially abundant whenever *E. aedis* was present (Fig. 1D). No equivalent spots were observed for *V. culicis* at the intersection of the V and VE treatments. This suggests the specific host response associated with *V. culicis* was altered to a greater extent by the presence of *E. aedis* than the specific host response to *E. aedis* by the presence of *V. culicis*. The specific responses to each of the parasites were temporally heterogeneous in different ways. The proteome of *Ae. aegypti* infected with *V. culicis* had a greater number of spots with modified abundance on day 5 relative to day 15 (compare treatments V and VE of Fig. 1A to Fig. 1B and C). In contrast, *Ae. aegypti* infected with *E. aedis* had a greater number of spots with modified abundance on day 15 (compare E and VE treatments in the same figures).

We can also consider spots that have abundance profiles shared between the single infection treatments and may be characteristic of a more general response to infection. These are illustrated by spots found in the zone where the two individual infections (V and E) intersect in Figure 1. A total of 27 spots were found in this area, including seven responding to all three infection treatments. All 27 proteins common to these treatments were differentially abundant on day 5 of the experiment, with five of them also being differentially abundant on day 15, but none were only differentially abundant on day 15 only. This pattern indicates that the host response directed toward each parasite shared more in common at five days postinfection that at 15 days postinfection. This trend could reflect the increasing divergence in the challenges posed by each infection as their respective infections progress beyond gut tissues and exploit different host tissues for different purposes.

#### The host response to coinfections compared to individual infections

Coinfections could represent a novel challenge to the host immune system beyond the sum of their individual infections. Figure 1D shows five proteins in the zone exclusive to the treatment where mosquitoes were challenged with both parasites. Apart from the seven spots modified in all infection treatments, there were 16 spots in the zone where the E and VE treatments intersect, but none in the zone where the V and VE treatments intersect (Fig. 1D). This indicates host response directed toward coinfections shared more in common with single infections involving *E. aedis* than single infections involving *V. culicis*. Furthermore, the similarity of the response to the E and VE treatments increased between days 5 and 15, as their intersection initially involved nine of 16 (Fig. 1C) proteins and rose to 16 of 16 for day 15 (Fig. 1B). Hence, although some of the host response was specific toward the coinfection condition, there was a bias suggesting the response to coinfection was more strongly influenced by the presence of *E. aedis*, and that its influence on the host response grew with time since infection.

Fewer proteins were modified by the coinfection treatment than each of the single infection treatments. A total of 28 proteins showed differential abundance in the zone described by the VE treatment, while those for the *E. aedis* and *V. culicis* treatments were 47 and 50, respectively (Fig. 1D). Thus, rather than suggesting the coinfection condition provoked a more diverse host response, it appears to have stimulated the modified abundance of fewer proteins. This could be because the host invested more heavily in a limited number of proteins with a general role in coping with infection. Alternatively, it could be that the coinfection inhibited the host's ability to respond to infection to a greater extent than to either individual infection.

#### Overall trends in patterns of protein abundance

The results demonstrate that *Ae. aegypti* responded to the different types of microsporidian infection with a range of proteins that were mainly specific to particular infection conditions. Certain proteins were elicited only in response to infection with *V. culicis* or *E. aedis*, while for others changes in abundance depended on the presence of the other parasite. The observed patterns suggested the response to *V. culicis* and *E. aedis* diverged with time postinfection, while the response to the coinfection and *E. aedis* tended to converge with time postinfection. The overall response to infection involved the modified abundance of fewer proteins in older infections and fewer proteins in response to the coinfection treatment than observed for single infections involving either parasite.

### Proteins identified

**Figure 2:**
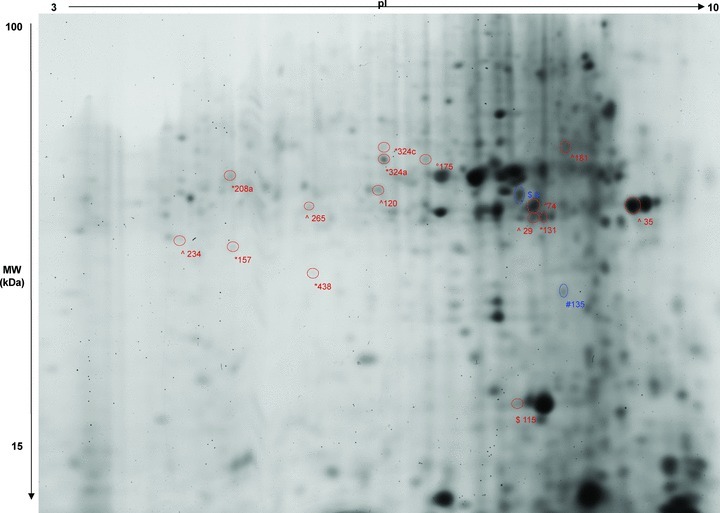
A representative 2D electrophoresis Coomassie-stained gel for protein extracted from infected larvae (replicates comprised of 10 pooled larvae from each treatment, with 26 µg of sample loaded on to each gel). The spot numbers indicated correspond to the proteins identified in Table 1 with modified abundance in the infection treatments. Protein spots shown in red were upregulated, and those in blue downregulated, relative to the uninfected control. Infection treatments with differential protein abundance on the different sampling days are indicated as follows *the single *V. culicis* treatment on day 5; **∧** the single *V. culicis* and the single *E. aedis* treatments on day 5; **°** the single *V. culicis* and the single *E. aedis* treatments on day 5 and day 15; **$** the single *E. eadis* treatment on day 15 and the coinfection treatment on day 15; and **#** the coinfection treatment on day 15 infection.

We identified some of the proteins involved in the host response to infection by picking the corresponding spots, followed by a MALDI-TOF MS analysis after a tryptic digestion (Fig. 2). The data from MS analysis were compared to records in the SwissProt and TrEMBL Insecta databases. A total of 17 proteins were identified (Table 1), 15 of which could be assigned an identity. In most cases, these involved proteins recovered from larvae sampled at five days postinfection and the volume of their spots was less than for uninfected individuals. They were also biased toward treatments involving single infections with *V. culicis* (14) or *E. aedis* (nine), with only three being associated with the coinfection treatment. Thus, the proteins identified only provide a partial picture of the proteins associated with the different infection conditions and how they varied over time. A more detailed treatment of each protein is given below.

**Table 1:** Proteins identified in this study.

Spot	Protein name	Access number^1^	MASCOT score^2^	Theoretical mass	Theoretical pI	Sequence coverage (%)	Peptide number	Day(s)	Treatment(s) involved^3^	Spot volume^4^
120^5^	Ferritin heavy chain-like protein	Q8T4R8_AEDAE	78	23,802	5.64	33	8	5	E, V	-
157	Catalase	Q1HRH7_AEDAE	92	48,836	7.28	27	10	5	V	-
265	Catalase	Q1HRH7_AEDAE	100	48,836	7.28	26	10	5	E, V	-
438	Catalase	Q1HRH7_AEDAE	88	48,836	7.28	24	9	5	V	-
9	Actin	Q17KG3_AEDAE	127	42,148.9	5.22	39	15	15	E, VE	+
29	Actin	Q17KG3_AEDAE	60	42,148.9	5.22	23	11	5	E, V	-
74	Actin	Q17KG3_AEDAE	53	42,148.9	5.22	21	8	5	V	-
115	Actin	Q178B0_AEDAE	67	41918.8	5.29	20	9	15	E, VE	-
120^5^	Vacuolar ATPase B subunit	Q9XYC8_AEDAE	96	55,466.3	5.38	27	10	5	E, V	-
175	Enolase	Q17KK5_AEDAE	51	46,877.1	6.28	21	9	5 and 15	E, V	-
324^1^	Enolase	Q17KK5_AEDAE	161	46,877.1	6.28	44	19	5	V	-
324^3^	Enolase	Q17KK5_AEDAE	104	46,877.1	6.28	30	10	5	V	-
234	V-type proton ATPase catalytic subunit A	VATA_AEDAE	52	68,527.6	5.26	11	6	5	E, V	-
131	Glutathione S-transferase	Q16P79_AEDAE	149	27,034	5.24	50	16	5	V	-
135	Arginine or creatine kinase	Q1HR67_AEDAE	152	40,191	5.97	38	14	5 and 15	VE	+
208^1^	Arginine or creatine kinase	Q1HR67_AEDAE	78	40,191	5.97	30	9	5	V	-
35	—	Q17G18_AEDAE	165	23,677	4.71	46	13	5	E, V	-
181	—	Q16YP3_AEDAE	84	29,644	4.89	35	8	5	E, V	-

1 “Access number” refers to accession number in SwissProt and TrEMBL protein databases.

2 “MASCOT scores” > 50 indicate extensive homology while those > 68 indicate significant identity (*P* < 0.05).

3 V, *V. culicis* only; E, *E. aedis* only; VE, coinfection with *V. culicis* and *E. aedis*.

4 Spot volume relative to uninfected larvae on same day(s) of sampling; + = higher, − = lower.

5 Spot 120 was assigned two possible identities.

### Ferritin

This protein has an important role in the transport and storage of iron within cells and in regulating the activity of reactive oxygen species (ROS). Relative to uninfected larvae, it was less abundant in the V and E treatments of larvae sampled on day 5. Changes in ferritin expression have been reported in a number of studies involving insects subject to infection from a diversity of pathogens (e.g., Levy et al. 2004; Vierstraete et al. 2004; Paskewitz and Shi 2005; Kremer et al. 2009; Masova et al. 2010), to which the microsporidia can now be added.

### Catalase

The main role of this protein is to catalyze the breakdown of hydrogen peroxide (H_2_O_2_) into water and oxygen, giving it an important role in the response to oxidative stress. Other studies have found catalase expression modified in infected insects (e.g., Ha et al. 2005; Tchankouo–Nguetcheu et al. 2010). In this experiment, the protein was less abundant in the V and E treatments of five-day-old larvae compared to uninfected larvae. It was also found in different isoforms, suggesting posttranscriptional modification. In the case of *Anopheles gambiae*, it has been suggested the host actively switches off its catalase production in response to blood meals containing *Plasmodium berghei*. This leads to locally elevated levels of H_2_O_2_ within the mid-gut as the meal is digested; these raised levels of H_2_O_2_ are toxic for the host and increase their rates of mortality; however, they are even more toxic for the invading pathogen and substantially reduce its chances of successfully establishing infection in tissues of the host gut wall (Molina–Cruz et al. 2008).

### Actin

Isoforms of this protein were found to be more or less abundant in different infection treatments and on both days of sampling (Table 1). It was also one of only two proteins identified with modified abundance in response to the coinfection treatment. Actin is a major component of the cytoplasm and cytoskeleton in eukaryotic cells and has many functional roles. Infected insects frequently show altered patterns of actin expression (e.g., Vierstraete et al. 2004; Kumar and Paily 2008; Mendes et al. 2008; Manalil et al. 2009; Tchankouo–Nguetcheu et al. 2010), not least because it is actively involved in deforming cellular structures during phagocytosis. However, its modified expression could also be due to its role in the repair of tissues damaged by invading pathogens (e.g., Gupta et al. 2005; Paskewitz and Shi 2005). Another reason why actin could be associated with a microsporidian infection is that host organelles, notably mitochondria and endoplasmic reticulum, are often found drawn into close proximity to developing parasites and actin will be involved in this intracellular reorganization.

### Vacuolar ATPase B subunit and V-ATPase subunit A

In five-day-old larvae of the V and E treatments, these proteins were significantly less abundant compared to uninfected larvae. These proteins are known for their role in pumping H^+^ ions across plasma membranes of cells or organelles within cells, using energy from the hydrolysis of ATP, which leads to the acidification of intracellular compartments. They also regulate the movement of some intracellular pathogens in and out of host cells, for example, enveloped viruses (Perez and Carrasco 1994). The downregulation of an ATP synthase beta subunit has been reported for *Ae. aegypti* infected by the dengue type 2 virus (Tchankouo–Nguetcheu et al. 2010). Many intracellular parasites develop inside parasitophorous vacuoles formed within host cells and some parasites cross these membranes with the help of vacuolar ATPases (e.g., Shahabuddin and Pimenta 1998; Huang et al. 2006). The plasma membrane of developing microsporidia can be in direct contact with the surrounding cytosol of its host cell or physically separated from it by an envelope derived from materials originating from the host, the parasite, or both (Cali and Takvorian 1999). The functional significance of these differences is currently unknown. Our results suggest vacuolar ATPases could be involved at these interfaces and directly implicated in the interaction between developing parasites and their host environment.

### Enolase

The primary role of enolase is as a glycolytic enzyme and its main activity is in the metabolism of carbohydrates, however, is has secondary roles including those in organizing the cytoskeleton and as a heat-shock protein (Pancholi 2001). It was found in two isoforms following the infection of *Drosophila melanogaster* with a fungus or two different types of bacteria (Levy et al. 2004), and in this study, it was also found in isoforms for both five- and 15-day-old larvae in the V and E treatments, where they were significantly less abundant. A study comparing the lipid, glycogen, and sugar content of five-day-old *Ae. aegypti* larvae infected or uninfected with *V. culicis* showed the infection to be energetically demanding for its host as infected larvae had significantly less of each resource (Rivero et al. 2007).

### Glutathione S-transferase

The glutathion S-transferases (GSTs) are a major group of detoxifying enzymes against a broad range of toxins, including insecticides. They are involved in the response to oxidative stress and their production can be induced by ROS. A lower abundance of GST in five-day-old larvae infected with *V. culicis* of this experiment was also found in a previous proteomic study involving *Ae. aegypti* and *V. culicis* (Biron et al. 2005). Other insects have also been found to modify the expression of their GSTs when infected (e.g., Vierstraete et al. 2004; Paskewitz and Shi 2005; Masova et al. 2010; Tchankouo–Nguetcheu et al. 2010). In addition, a study on the ability of *P. berghei* to infect *An. gambiae* found silencing the expression of two GSTs significantly reduced the parasite's infection success (Jaramillo–Gutierrez et al. 2009), confirming these proteins have a role in Dipteran responses to infection.

### Arginine/creatine kinases

These proteins help regulate the energy metabolism of cells in catalyzing the formation of ATP from ADP (or vice versa) and act as transporters of energy between sites of its production and utilization by ATPases. Two spots corresponding with arginine or creatine kinase were found with modified abundance in this study, one showed an increase in abundance relative to uninfected individuals on both days 5 and 15 in response to the coinfection (VE) treatment (Table 1). This is interesting because increased expression of arginine kinase has been reported for other Diptera exposed to infection (e.g., Levy et al. 2004; Vierstraete et al. 2004; Tchankouo–Nguetcheu et al. 2010), and silencing its expression reduced the infection success of *P. berghei* and *P. falciparum* in *An. gambiae* (Jaramillo–Gutierrez et al. 2009). The latter result indicates an increase in this type of metabolic activity could be provoked by invading pathogens for their own benefit, rather than by hosts in their own defense.

## Conclusion

The results of this study provide insights into how the proteome of *Ae. aegypti* responded to infections involving two different microsporidian parasites and that of their coinfection. Each of the 75 proteins described in Figure 1 showed significantly modified abundance in response to at least one of the infection treatments on at least one of the two days when host larvae were sampled. This equates to approximately 10% of the 646 spots reliably detected and does not include proteins whose abundance was modified in response to hypoxia.

Some proteins only showed modified abundance in a particular infection treatment and time of sampling, whereas other proteins were differentially abundant in all infection treatments and at both times of sampling, relative to that recorded from uninfected larvae. This indicates sensitivity and flexibility in the host proteome in response to the infection conditions it encounters and how they develop with time since infection.

The host response toward individual infections of each parasite shared more in common for larvae sampled on day 5 than for those taken on day 15. This result may reflect that the two parasites initially exploit the same host tissues (host cells lining the mid-gut) before diverging in the types of tissues they exploit. This could cause an increasing divergence in the challenge posed by each type of parasite as their development continues. The host response in the coinfection treatment shared more in common with the response to *E. aedis* than for *V. culicis*, and this similarity increased in the older larvaeFAQ. Both of these results indicate the dynamic nature of the challenge posed by each type of infection and the host's deployment of proteins in response.

The observation that the host response to the coinfection treatment has more in common with the single *E. aedis* infection treatment corroborates a previous experiment investigating the effects of single and coinfections on mosquito and parasite life history (Duncan et al., unpubl. ms.). This experiment found that single *E. aedis* infections were more virulent than single *V. culicis* infections, and that coinfections affected host life-history traits in a similar way to single *E. aedis* infections.

Finally, fewer proteins were involved in response to coinfection than toward either single infection. This could indicate the host concentrated its response in a narrower range of proteins with more general utility, rather than dispersing its resources over a broader range of proteins with more specific roles in maintaining its condition. It may also be that individual responses to each parasite may be canceled out, or the host immune system may be constrained and not able to mount a response that is greater than either of the single infections. Alternatively, if the host proteome response reflects parasite manipulation of the immune system a smaller response may reflect inhibition of a parasite's manipulation in the presence of a competitor. Such responses could be important in determining the strength of selection acting against different types of parasite in single versus multiply infected hosts.

The only published study we know of comparing immunity in single versus multiple infections used very different tools (Wegner et al. 2009). They established that the response to single strain infections was governed by additive, dominance, and epistatic components, while the response to multiple strain infections was driven by maternal components. This suggests a major difference in the architecture of the response to single versus multiple strain infections. Our results parallel these conclusions to some extent in that we show that the host proteome reaction to single infections was generally different to the response to coinfection.

As with every technique, 2D gel separation has its own limitations. In particular, it is only able to detect proteins within a range of 15–100 kDA. Although many immune-related proteins fall within this range (Iwanaga and Lee 2005), some of the smaller antimicrobial peptides, such as cecropins and cationic peptides important for antifungal activity, are too small to be detected (Vizioli and Salzet 2002). Thus, our ability to detect specific immune-related proteins was limited by both the size and magnitude of differential abundance. Importantly, the proteins we identified have been previously observed to serve a role against immune challenged mosquitoes or other Dipteran species.

Some of the proteins we identified, such as actin and ferritin, are frequently cited, thus indicating their importance in protecting hosts against a diverse range of pathogens. Other proteins are less frequently cited, indicating a more specific interaction between the host and the particular organism invading it, for example, reports implicating vacuolar ATPases are biased toward studies involving obligate intracellular pathogens. This gives some insight into the constraints associated with this type of life style and provides a focal point for further studies involving this type of pathogen.

Many vector-borne diseases involve pathogens whose development is partially, if not wholly, intracellular. Many bacteria and viruses use various mechanisms to prevent their host cell from being coinfected by rival strains or pathogens (Marschall et al. 1997). Thus, the ability of such pathogens to colonize a vector can depend on whether its cells are already occupied by another pathogen or not. The consequences of one intracellular pathogen reducing the chances of a vector becoming coinfected by another could be useful in reducing disease transmission *Wolbachia* have been proposed as a means of reducing the transmission of dengue and other viruses by mosquitoes (Moreira et al. 2009; Bian et al. 2010). These studies have shown the virus has only limited ability to colonize cells already occupied by the bacteria (Moreira et al. 2009). Furthermore, the presence of an established infection could "prime" the host immune system and make conditions much less favorable for the infection success of subsequent pathogens (Moreira et al. 2009). This has been suggested as one of the reasons as to why *Plasmodium* spp. experience reduced infection success in mosquitoes already infected by *V. culicis* (Bargielowski and Koella 2009).

The microsporidia are considered as candidates for reducing the transmission success of human pathogens vectored by mosquitoes (Koella et al. 2009). The chances of mosquitoes reaching adulthood are often reduced if microsporidia infect them as larvae (Agnew et al. 2003). Infected females surviving to adulthood are less likely to take blood meals (Koella and Agnew 1997) thus reducing their exposure to blood-borne pathogens, and the subsequent chances of transmitting them. The production of microsporidian spores can damage mosquito gut tissues and reduce the infection success of subsequent pathogens (e.g., *Plasmodium*; Bano 1958), and their infections may "prime" the host immune system (Biron et al. 2005; Bargielowski and Koella 2009) making the host environment more hostile for other pathogens. Finally, this study found microsporidian infections modified the abundance of host vacuolar ATPases. These proteins are involved in the establishment of intracellular infections, including those of *Plasmodium* and dengue (Perez and Carrasco 1994; Huang et al. 2006). This indicates another means by which microsporidian infections could disrupt the infection and/or transmission success of other pathogens trying to coinfect the same mosquito.

## Appendix A1

**Table:** Summary of infection levels for *V. culicis* and *E. aedis* in all treatment groups where infection could be confirmed.

	*Vavraia culicis*									*Edhazardia aedis*							
	Single *V. culicis*				Mixed infection				Single *E. aedis*				Mixed infection			
	No hypoxia		Hypoxia		No hypoxia		Hypoxia		No hypoxia		Hypoxia		No hypoxia		Hypoxia	
	Day 5	Day 15	Day 5	Day 15	Day 5	Day 15	Day 5	Day 15	Day 5	Day 15	Day 5	Day 15	Day 5	Day 15	Day 5	Day 15
Proportion infected, (± CI)	-	94%, (−0.814, +0.984)	-	72%, (−0.573, +0.832)	-	86%, (−0.713, +0.939)	-	0.57, (−0.422, +0.709)	86%, (0.706, +0.937)	86%, (−0.713, +0.939)	89%, (−0.740, +0.955)	88%, (−0.750, +0.948)	91%, (−0.787, +0.972)	87%, (−0.711, 0.972)	89%, (−0.759, +0.958)	88%, (−0.700, +0.958)

## Appendix A2

**Table:** Summary of protein spots with abundance profiles affected by experimental treatments, but not included in analyses as differences are not attributable to infection treatments, or did not show clear patterns in the change of abundance.

	Sampling day only	Hypoxia alone, or combined with sampling day or parasite treatment	Influence of parasite treatment	Combined influence of infection and sampling day
Number of spots	176	51	8	13
Reason excluded	Differences in abundance profiles unrelated to infection treatments.	Abundance depends on stress induced by hypoxia treatment, and not directly on presence of infection.	Differences in abundance expression profiles could not be detected between infected and uninfected larvae (e.g., spot volume was different between two parasite treatments, but neither was different from uninfected larvae).	Heterogeneous patterns of abundance on the two sampling days (e.g., increased volume on day 5 in response to infection with *V. culicis*, but decreased volume on day 15 in those infected by *E. aedis*).
Observations and remarks	Spots sampled on day 15 were larger than spots sampled on day 5.	Spots from day 5 tended to be smaller.		Although these proteins may be active in the response to infection, their exclusion avoids overinterpretation of abundance.
